# Circulating Immunoglobulins Are Not Associated with Intraplaque Mast Cell Number and Other Vulnerable Plaque Characteristics in Patients with Carotid Artery Stenosis

**DOI:** 10.1371/journal.pone.0088984

**Published:** 2014-02-21

**Authors:** Sanne Willems, Daniël van der Velden, Paul H. A. Quax, Gert Jan de Borst, Jean-Paul P. M. de Vries, Frans L. Moll, Johan Kuiper, René E. M. Toes, Saskia C. A. de Jager, Dominique P. V. de Kleijn, Imo E. Hoefer, Gerard Pasterkamp, Ilze Bot

**Affiliations:** 1 Laboratory of Experimental Cardiology, University Medical Center Utrecht, Utrecht, The Netherlands; 2 Division of Biopharmaceutics, Leiden Academic Centre for Drug Research, Gorlaeus Laboratories, Leiden, The Netherlands; 3 Department of Surgery, Leiden University Medical Center, Leiden, The Netherlands; 4 Einthoven Laboratory of Experimental Vascular Medicine, Leiden University Medical Center, Leiden, The Netherlands; 5 Department of Vascular Surgery, University Medical Center Utrecht, Utrecht, The Netherlands; 6 Department of Vascular Surgery, Sint Antonius Hospital, Nieuwegein, The Netherlands; 7 Department of Rheumatology, Leiden University Medical Center, Leiden, The Netherlands; 8 Interuniversity Cardiology Institute of the Netherlands (ICIN), Utrecht, The Netherlands; 9 Cardiovascular Research Institute and Surgery, National University Hospital Singapore, Singapore, Singapore; University Heart Center Freiburg, Germany

## Abstract

**Background:**

Recently, we have shown that intraplaque mast cell numbers are associated with atherosclerotic plaque vulnerability and with future cardiovascular events, which renders inhibition of mast cell activation of interest for future therapeutic interventions. However, the endogenous triggers that activate mast cells during the progression and destabilization of atherosclerotic lesions remain unidentified. Mast cells can be activated by immunoglobulins and in the present study, we aimed to establish whether specific immunoglobulins in plasma of patients scheduled for carotid endarterectomy were related to (activated) intraplaque mast cell numbers and plasma tryptase levels. In addition, the levels were related to other vulnerable plaque characteristics and baseline clinical data.

**Methods and Results:**

OxLDL-IgG, total IgG and total IgE levels were measured in 135 patients who underwent carotid endarterectomy. No associations were observed between the tested plasma immunoglobulin levels and total mast cell numbers in atherosclerotic plaques. Furthermore, no associations were found between IgG levels and the following plaque characteristics: lipid core size, degree of calcification, number of macrophages or smooth muscle cells, amount of collagen and number of microvessels. Interestingly, statin use was negatively associated with plasma IgE and oxLDL-IgG levels.

**Conclusions:**

In patients suffering from carotid artery disease, total IgE, total IgG and oxLDL-IgG levels do not associate with plaque mast cell numbers or other vulnerable plaque histopathological characteristics. This study thus does not provide evidence that the immunoglobulins tested in our cohort play a role in intraplaque mast cell activation or grade of atherosclerosis.

## Introduction

The incidence of atherosclerotic disease is increasing by the aging population and the life style in the Western world. The mast cell, a prominent inflammatory cell type and a major effector cell in allergy and asthma, has been shown to accumulate both in the rupture-prone shoulder region of human atheromas (1,2) and in the perivascular tissue during atherosclerotic lesion progression (3). Recently, we have shown that intraplaque mast cell numbers are associated with plaque vulnerability and interestingly, with future cardiovascular events (4). In that study, mast cells numbers associated with vulnerable plaque characteristics such as lipid core size, intraplaque haemorrhage, microvessel density and inflammatory cell accumulation, suggesting that mast cells actively contribute to atherosclerotic plaque progression and destabilization. Inhibition of mast cell activation may therefore be of interest for future therapeutic interventions. However, the mechanism of mast cell activation during the development of atherosclerosis remains up to date unresolved. Previously, we and others have established that mast cells in the vessel wall can be activated by for example neuropeptides (5), complement factors (6) and lipid mediators (7) in animal models of atherosclerosis. Furthermore, the mast cell expresses the high-affinity IgE receptor (FcεR1) and the IgG receptor (FcγR) (8,9). Mast cells can be activated via IgE mediated crosslinking of the FcεR, after which mast cells release granules into the surrounding area. IgE levels have been shown to be elevated in patients with unstable angina pectoris (10) and intriguingly, also higher in dyslipidemic men as compared to control subjects (11). Furthermore, Lappalainen et al demonstrated that specific oxLDL-IgG immune complexes were able to induce mast cell activation (12). Circulating specific IgE and IgG antibodies or lipid-immunoglobulin immune complexes, which exert their effects through the FcεR and FcγRs, are known to play a role in several immune responses (9) and may thus also be involved in mast cell activation within the atherosclerotic plaque, thereby affecting plaque stability. Based on these observations, we hypothesize that circulating immunoglobulins may be involved in or be reflective of mast cell activation and thereby accelerate the destabilization of the atherosclerotic plaque. This study was designed to assess the presence of associations between immunoglobulin expression and mast cell numbers in plaques from patients with carotid stenosis. Hence, we assessed total and ox-LDL specific IgG and total IgE plasma levels and related their numbers to several mast cell parameters and established vulnerable plaque characteristics. In additions, immunoglobulin levels were related to clinical characteristics.

## Materials and Methods

### Study Population and Design

A total of 135 patients of the Athero-Express were included in this study. The Athero-express biobank involves patients that underwent carotid endarterectomy (CEA) in two Dutch teaching hospitals in Utrecht and Nieuwegein the Netherlands (13). The criteria to perform carotid endarterectomy were based on the recommendations by the Asymptomatic Carotid Atherosclerosis Study (ACAS study) for asymptomatic patients and the North American Symptomatic Carotid Endarterectomy Trial and the European Carotid Surgery Trial (NASCET study) for symptomatic patients (14–18). Patients were operated between March 2002 and August 2008 of which intraplaque mast cell numbers were available (4). In that study, patients were selected who remained healthy and patients who suffered from an event during follow-up in a 2∶1 ratio. Of 135 patients blood plasma samples were available. Total mast cell numbers and baseline characteristics did not differ between the patients of which plasma was and was not available. The local medical ethical boards of both participating hospitals approved this study. The participating patients signed a written informed consent prior to inclusion. The patient’s baseline characteristics and medical history were obtained via questionnaires and the patient medical records.

### Materials

The carotid plaques used in this study were processed as described previously (13). In short, after surgical dissection the plaque was cut into segments of 5 mm. The segment with the largest plaque area was fixed in formalin and embedded in paraffin for histology. The two adjacent sections were frozen in liquid nitrogen and used for protein isolation. In addition, blood was drawn prior to CEA procedure and plasma was stored at −80°C.

### Quantification of Immunoglobulin and MC Tryptase Levels in Patient Plasma

Plasma total IgE and IgG levels were measured using a human IgG and IgE ELISA according to manufacturer’s protocol (Bethyl Laboratories, Montgomery, TX). Plasma IgG-oxLDL levels were measured by coating cupper oxidized human LDL in PBS (pH = 9,0) on MaxiSorp 96-well plates (Nunc, Roskilde, Denmark) overnight at 4°C. Diluted Samples and standards (Biomedica, Wien) were added and incubated for 2 hours at 37°C. Supernatants were discarded and plates were washed thoroughly. Anti-human IgG-HRP (Bethyl Laboratories, Montgomery, TX) was added as detection antibody for 1 hour at 37°C. Bound oxLDL-IgG was visualized by using 2,2′-azinobis 3-ethylbenzthiazoline-6-sulfonic acid (ABTS, Sigma). Colour was measured at an optical density of 415 nm using a conventional ELISA reader. Between each ELISA step plates were washed with PBS containing 0.05% Tween20.

MC tryptase levels were determined in plasma samples using an ImmunoCAP**®** 250 tryptase assay (Phadia AB, Uppsala, Sweden).

### Immunohistochemistry

Sections were stained for mast cell tryptase (mast cells), CD68 (macrophages), smooth muscle cells (alpha actin), and CD34 (endothelial cells) as previously described (4). Total mast cell numbers were determined by counting all (degranulating) mast cells present in a plaque cross-section at ×40 magnification (4). A degranulating mast cell was defined by a group of mast cell tryptase positive extracellular granules in close proximity of each other or in close proximity of a mast cell. The total plaque area (mm^2^) was measured using the analySIS 2.8 software (Olympus Soft Imaging Solutions GmbH, Münster, Germany) to determine the distribution density of mast cells expressed as numbers of mast cells/mm^2^. Image-analyzing software was used to determine positive macrophage and smooth muscle cell staining expressed as a percentage of covered plaque area (13). Microvessels were counted in three hot-spots and were expressed as average microvessel density per hotspots (19). Collagen content (picrosirius red) was scored semi-quantitatively. The size of the extracellular lipid core (atheroma) was assessed by the H&E and picrosirius red stain (13).

### Statistics and Data Analysis

IBM SPSS statistics version 20 was used for all analyses (IBM corporation, Armonk, NY, USA). Immunoglobulin levels are not normally distributed; non-parametrical testing was used to determine differences. The Mann-Whitney U test was used to study immunoglobulin levels as a continuous variable for all risk factors. The Spearman correlation coefficient was calculated to assess associations between immunoglobulin levels and all continuous variables in this study. Differences were considered significant with a p-value of below 0.05.

## Results

### Baseline Patient Characteristics

Total IgE, total IgG and oxLDL-IgG plasma levels were measured in a total of 135 patients that underwent carotid endarterectomy. Baseline clinical characteristics of the 135 patients are provided in Table 1. The studied patient population with a mean age of 67 and a male prevalence (71%) reflects a relatively typical population of patients with vascular occlusive diseases. The majority of patients was symptomatic (74%) as illustrated by the incidence of amaurosis fugax, a TIA or a stroke, was hypertensive (86%) and used statins (69%).

**Table 1:** Baseline characteristics of patients in relation to immunoglobulin plasma levels.

		Total IgG	p-value	oxLDL-IgG	p-value	Total IgE	p-value
Age, mean years (sd)	67 (9)	r = −0.082	0.347	r = 0.031	0.724	r = 0.097	0.265
BMI, mean kg/m^2^ (sd)	27 (4)	r = −0.115	0.19	r = 0.001	0.995	r = −0.126	0.15
Gender							
Male	96/135 (71%)	16.8 [11.7–23.5]	0.694	336 [239–499]	0.074	131.1 [57.5–317.4]	0.169
Female	39/135 (29%)	17.2 [12.6–23.1]		282 [233–358]		76.7 [31.4–293.4]	
Current smoker							
Yes	55/134 (41%)	16.6 [10.3–23.3]	0.635	303 [252–481]	0.427	173.4 [65.5–449.8]	**0.032**
No	79/134 (59%)	17.1 [12.3–23.2]		306 [219–502]		91.5 [41.5–203.0]	
Diabetes mellitus							
Yes	25/135 (19%)	15.4 [12.1–22.9]	0.63	288 [241–391]	0.554	79.8 [41.9–288.5]	0.329
No	110/135 (81%)	17.0 [12.3–23.7]		320 [238–489]		125.0 [57.3–312.1]	
Statin Use							
Yes	93/135 (69%)	17.1 [12.3–22.8]	0.72	288 [224–406]	**0.004**	97.5 [43.1–276.8]	**0.012**
No	42/135 (31%)	16.8 [23.3–25.0]		399 [282–584]		157.3 [75.2–545.4]	
Hypertension							
Yes	116/135 (86%)	17.2 [12.5–23.2]	0.633	302 [233–480]	0.207	113.0 [49.1–304.8]	0.265
No	19/135 (14%)	15.0 [10.4–26.0]		341 [260–537]		183.3 [68.8–594.0]	
Hypersensitive							
Yes	27/132 (20%)	17.1 [12.6–22.2]	0.906	305 [233–412]	0.539	148.1 [44.6–449.8]	0.401
No	105/132 (80%)	16.6 [11.6–23.9]		303 [237–489]		115.6 [50.5–304.7]	
History VI							
Yes	54/135 (40%)	18.5 [11.9–23.5]	0.39	297 [216–483]	0.391	86.2 [47.7–274.4]	0.239
No	81/135 (60%)	15.4 [11.9–23.5]		306 [254–484]		135.4 [54.2–358.9]	
History MI							
Yes	30/134 (22%)	19.0 [12.7–22.7]	0.673	321 [222–428]	0.62	85.6 [51.7–282.9]	0.601
No	104/134 (78%)	16.5 [12.2–23.5]		304 [239–508]		129.7 [49.1–310.8]	
Clinical presentation							
Asymptomatic	35/135 (26%)	15.8 [12.3–23.2]	0.419*	314 [239–505]	0.377*	113.0 [47.0–304.9]	0.431*
Symptomatic	100/135 (74%)	18.1 [12.1–24.1]		293 [224–399]		144.3 [68.8–356.7]	
Amaurosis fugax	22/135 (16%)	16.6 [12.5–24.9]		296 [240–424]		120.9 [46.3–327.8]	
TIA	51/135 (38%)	16.2 [11.5–23.2]		300 [223–512]		106.3 [32.9–281.6]	
Stroke	27/135 (20%)	15.4 [12.3–22.7]		352 [257–515]		135.4 [66.6–319.9]	

Data are presented as No. (%) and median [IQR] unless otherwise indicated; r = Spearman’s rank correlation coefficient; sd = standard deviation; IQR = interquartile range; BMI = body mass index; TIA = transient ischemic attack; *p-value represents statistical analysis for asymptomatic patients versus symptomatic patients (composed of amaurosis fugax, TIA an stroke).

### Plasma Immunoglobulin Levels and Mast Cell Parameters

A correlation with borderline significance was found between total IgE and oxLDL-IgG (r = 0.169, p = 0.051). Total IgG did not correlate with either total IgE or oxLDL-IgG. No association was found between total IgE, total IgG and oxLDL-IgG plasma levels and total mast cell numbers or total degranulating mast cells (Table 2). In addition we did not observe any association between the three immunoglobulins and tryptase plasma levels.

**Table 2:** Immunoglobulin plasma levels with respect to mast cell parameters.

	Total IgG	p-value	oxLDL-IgG	p-value	Total IgE	p-value
Total mast cells	r = −0.038	0.664	r = 0.137	0.114	r = −0.038	0.664
Mast cells/mm^2^	r = −0.104	0.23	r = 0.115	0.186	r = −0.014	0.872
Degranulating mast cells/mm^2^	r = −0.074	0.519	r = 0.008	0.946	r = −0.076	0.506
Plasma tryptase	r = −0.076	0.378	r = 0.040	0.643	r = 0.064	0.457

Data are presented as Spearman’s rank correlation coefficient (r).

### Plasma Immunoglobulin Levels and Vulnerable Plaque Characteristics

**Figure 1:**
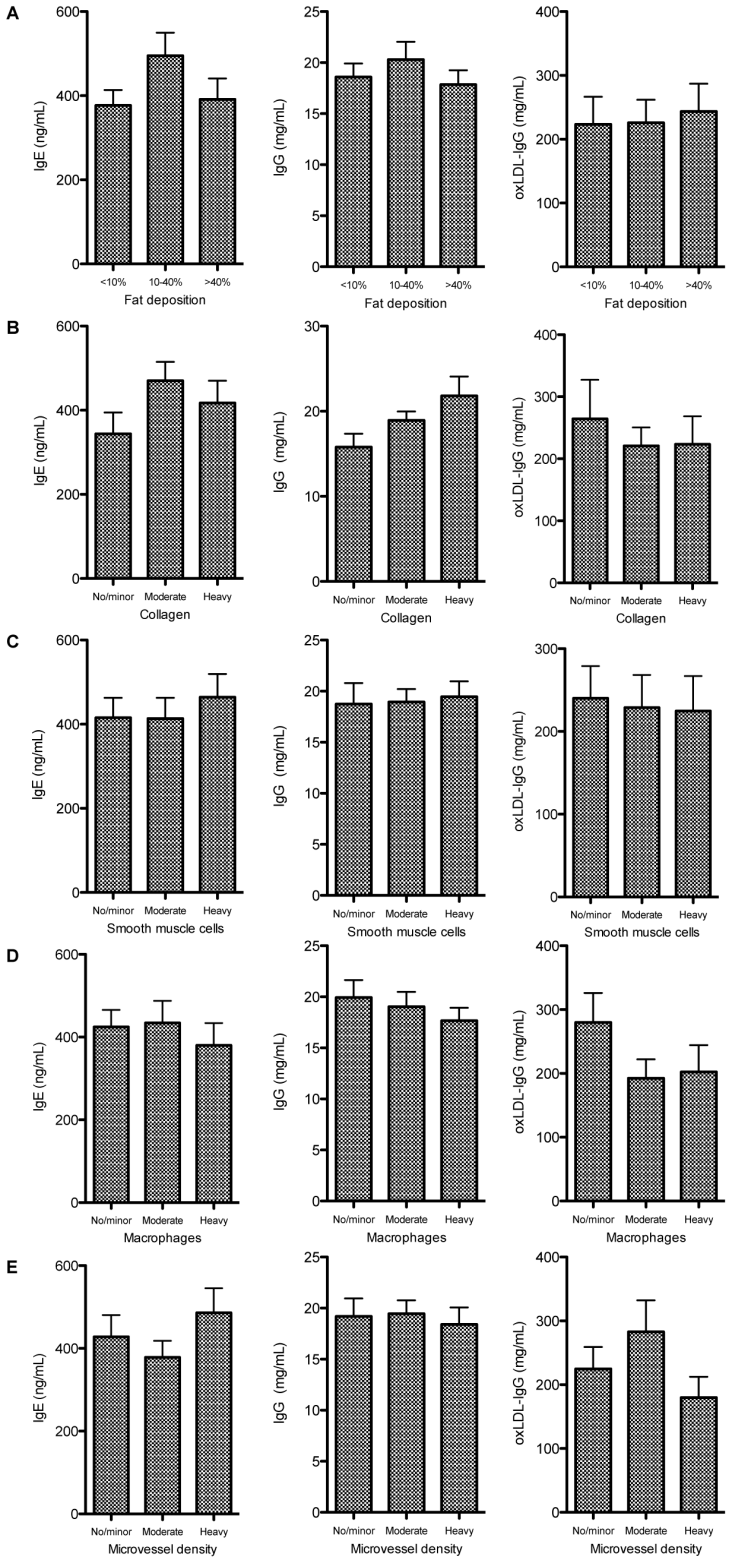
No association were found between IgE (left graphs), IgG (middle graphs) and oxLDL-IgG (right graphs) levels and any of the plaque characteristics: fat deposition, collagen, smooth muscle cells, macrophages and microvessel density.

As depicted in Figure 1, no consistent associations were found between immunoglobulin levels and measures of vulnerable plaque phenotype. There was no association between immunoglobulin levels and any of the following plaque characteristics: fat deposition, collagen, smooth muscle cells, macrophages and microvessel density.

### Plasma Immunoglobulin Levels and Clinically Relevant Characteristics

Table 1 provides an overview of the associations between the three immunoglobulin expression levels and relevant clinical characteristics. A negative association was observed between statin use and total IgE (97.5 [43.1–276.8] vs. 157.3 [75.2–545.4] ng/mL, p = 0.012) and oxLDL-IgG (288 [224–406] vs. 399 [282–584] mU/mL, p = 0.004). Higher total IgE levels were observed in smokers compared to non-smokers (91.8 [62.3–145.0] vs. 76.7 [47.1–111.2] ng/mL, p = 0.002). Clinical presentation was not associated immunoglobulin levels: no differences were observed in expression levels for any of the immunoglobulins between asymptomatic patients (n = 35) and symptomatic (n = 100) patients. Furthermore, we did not detect any differences in any of the circulating immunoglobulin levels between the subgroups of symptomatic patients (i.e. patients suffering from amaurosis fugax, TIA or stroke). Additionally, no association was found between immunoglobulin levels and the delay between surgery and presentation of symptoms.

## Discussion

Progression of an atherosclerotic plaque is often characterized by measures of plaque vulnerability. A vulnerable plaque is prone to rupture resulting in severe cardiovascular complications. Experimental studies have shown that mast cell activation results in progression and destabilization of the atherosclerotic plaque (20,21). In addition, in human plaques, mast cells correlated with vulnerable plaque characteristics and appeared associated with future combined cardiovascular events (4). It has been suggested that activated mast cells induce intraplaque neoangiogenesis thereby making the plaque more susceptible for rupture. For therapeutic intervention, identification of the endogenous mast cell activators during the progression and destabilization is of great value. In allergy and asthma, IgE is the main trigger for mast cell degranulation via crosslinking of the FcεR, while specific IgG immune complexes can exert mast cell activation resulting in cytokine release via binding to various Fcγ receptors. Previous studies have demonstrated that plasma IgE levels may be linked to the presence of cardiovascular diseases (11) and mice lacking the FcεR displayed reduced atherogenesis (22). Furthermore, oxLDL specific IgG molecules that can form immune complexes with oxLDL have been detected in human and rabbit atherosclerotic plaques (23) and these immune complexes were able to induce TNFα and IL-8 release from human mast cells (12). Taken together, these data suggest that IgE and specific IgGs may be important mast cell activators in cardiovascular disease.

It is however still unknown whether circulating immonuglobulins are capable of activating mast cells in human atherosclerotic plaques. Here we show that circulating IgG, IgE and oxLDL-IgG are not associated with mast cell determinants in a patient cohort with severe carotid stenosis that underwent an endarterectomy. No association was observed between any of the three immunoglobulins measured in this study and total plaque mast cell numbers or mast cell tryptase plasma levels. In addition, we did not observe any correlation with intraplaque degranulating mast cell numbers. Our results do not provide supportive evidence that increased immunoglobulin levels induce activation of mast cells in advanced human atherosclerotic plaques.

Previously, IgE was shown an independent marker for cardiovascular disease in men (24). Therefore we explored the possibility of an association between the three immunoglobulins had any future adverse events in patients with established cardiovascular disease. We did not find any association between immunoglobulin levels and future cardiovascular events, however, we were underpowered for analysing risk prediction for the immunoglobulins in our cohort. Nevertheless, no association was found with histological markers of plaque vulnerability, one of the most important determinants for future cardiovascular complications.

Serum oxLDL specific IgG antibodies have previously been linked to the presence and destabilization of the atherosclerotic plaque (25). As mentioned above, specific IgGs have been observed within the atherosclerotic plaque. In the current study, we did not detect any correlation between plasma oxLDL-IgG levels and mast cell activation or plaque phenotype. However, systemic oxLDL-IgG levels may not reflect the local oxLDL-IgG immune complexes that may actually activate the mast cell within the atherosclerotic plaque. Histological analysis of immune complexes, and with that also local IgE content, and colocalization with mast cells within the plaque may provide more information on the mechanisms of mast cell activation in atherosclerosis.

Interestingly, lower IgE and oxLDL-IgG levels were observed in patients that used statins. When we differentiated between the patients on statins and the patients not on statins it does not affect the outcome of the associations observed between immunoglobulin levels and mast cell parameters or plaque vulnerability (data not shown). The inhibitory effects of statin use on oxLDL-IgG antibody levels have been previously described (25), however reduced levels of IgE after statin treatment has to our knowledge not been reported before in patients with atherosclerosis. These data thus identify a novel effect of statin treatment, in addition to lipid-lowering, possibly by affecting antibody production of B cells.

The results of our study may be limited by the relative small number of patients. We have previously established that plasma tryptase levels of these patients correlate with plaque mast cell number and activation status, thus being reflective of mast cell activation. However, using these patients we were unable to detect a relation between any of the measured circulating immunoglobulin levels and intraplaque mast cell numbers or its activation, which may be improved by using a larger patient cohort. In addition, we measured plasma immunoglobulin levels at a single time-point that is at time of carotid endarterectomy, which is most reflective of the mast cell status within the plaque at that time point. Measuring plasma immunoglobulin levels at multiple time-points, for example at follow-up, may provide more information on the variation in circulating immunoglobulin levels together with disease progression.

In conclusion, no associations were found for total IgE, total IgG and oxLDL-IgG and the presence of total mast cell numbers or the number of degranulating mast cells in atherosclerotic plaques. Furthermore, the immunoglobulins were not related to most of the established characteristics of the rupture-prone atherosclerotic plaques. Taken together, this study does not provide supportive evidence that the three investigated circulating immunoglobulins activate mast cells during the progression of atherosclerotic disease. We can however not exclude that the mast cells may be activated by other specific immunoglobulins, or that local factors within the vessel wall are more predominant determinants of mast cell activation in the atherosclerotic plaque. Future research on the local environmental specific IgE and IgG levels within the plaque may thus shed more light on mechanism of mast cell activation in atherosclerosis.
